# Effects of Different Processed Diets on Growth Performance, Blood Parameters, Hair Quality and Fecal Microbiota in Ragdoll Cats

**DOI:** 10.3390/ani14182729

**Published:** 2024-09-20

**Authors:** Peng Wang, Xin Tian, Jie Feng

**Affiliations:** Key Laboratory of Animal Nutrition and Feed of Zhejiang Province, College of Animal Sciences, Zhejiang University, Hangzhou 310027, China; 3150100113@zju.edu.cn (P.W.); 22317028@zju.edu.cn (X.T.)

**Keywords:** meat diet, extruded dry food, feline nutrition, Ragdoll cats, hair quality, fecal microbiota

## Abstract

**Simple Summary:**

The dietary preferences for pet cats have been a topic of debate, with extruded dry food, cooked meat, and raw meat being the primary contenders. This study aimed to compare the effects of these three diets on growth performance, blood parameters, fecal scores, and fecal microbiota composition in purebred Ragdoll cats (*n* = 5/group). These data, to some extent, suggest that CM is the most suitable diet for Ragdoll cats, but further research on intestine microbiota is still needed. Our study result provides valuable insights for purebred pet cat breeding.

**Abstract:**

In recent years, there has been ongoing debate about the dietary choices for pet cats, particularly regarding three options: extruded dry food, cooked meat, and raw meat. Determining which diet is most suitable for a cat’s healthy growth still requires substantial empirical support. Our study aimed to evaluate the effects of feeding Ragdoll cats (*n* = 5/group) extruded dry food (ED), cooked meat (CM), and raw meat (RM) on their growth performance, apparent digestibility, fur condition, blood parameters, fecal scores, and gut microbiota composition. However, our results indicate that different types of diets did not significantly affect the daily weight gain of Ragdoll cats. The CM group showed a significant improvement in the digestibility of dry matter, fat and protein compared to the ED group (*p* < 0.05) but no improvement in that of fat compared to the RM group. Compared to the ED group, both the CM and RM groups showed significant improvements in fur condition while exhibiting a significant decrease in fecal scores (*p* < 0.05). The CM and RM groups exhibited enhanced serum antioxidant capacity (*p* < 0.05) and increased immunity in the cats (*p* < 0.05). Immunity enhancement in the CM group was significantly higher than that in the RM group(*p* < 0.05). The ED group showed an increase in the abundance of beneficial bacteria in Ragdoll cat intestines, while the CM and RM groups showed enhancements in the innate microbiota of feline animals. These data, to some extent, suggest that CM is the most suitable diet for Ragdoll cats, but further research on intestine microbiota is still needed. These study findings provide a reference for purebred pet breeding purposes.

## 1. Introduction

With the development of the economy, there has been a continuous rise in pet ownership in China. Many people recognize companion animals as their friends, partners, and family members. In comparison to dogs, cats are more suitable for urban living due to their cleanliness and ease of care. Consequently, the feline population is experiencing rapid growth. The global feline population has been rising steadily, with household cat numbers reaching approximately 1.1 billion in Europe, 95.6 million in the United States, and 48.6 million in China [1]. While many cats consume extruded dry food (ED), only a small proportion are fed canned food. Simultaneously, the concept that cats are naturally inclined towards raw meat (RM) has emerged, leading some pet owners to opt for RM diets [2]. Previous studies have highlighted the potential risk of microbial contamination associated with raw food [3,4,5]. ED is a complete feed produced using extrusion technology with low moisture content and balanced nutrition that offers convenient consumption options. Conversely, cooked meat (CM) is typically prepared through high-temperature cooking processes, which ensure high moisture content while also guaranteeing microbiological safety due to the sterilization process.

Research on the effects of food on cats is scarce. Kerr et al. investigated the effects of 1- to 3-day-old whole chicks, adult chicken products, chicken-based canned diets, and chicken-based extruded diets on apparent total tract energy and macronutrient digestibility, ME, nitrogen balance, and blood metabolites in domestic cats [6]. Vester et al. compared the effects of a high-protein extruded kibble diet and a commercial RM-based diet on apparent nutrient digestibility, fecal characteristics, nitrogen balance, and blood metabolite concentrations in captive African wildcats [7]. However, there has been limited research on the effects of different types of diets on purebred cats, such as Ragdoll cats.

The objective of this study was to compare the effects of three different types of processed diets—ED, CM, and RM—on growth performance, apparent digestibility of nutrients, fecal characteristics, blood biochemistry, hair quality, and fecal microbes in Ragdoll cats.

## 2. Materials and Methods

### 2.1. Diets

ED was a common and representative commodity feed purchased from the market. CM and RM feeds were manufactured at Jiangsu Yichong Biotechnology Co., Ltd. (Suqian, China). (CM was cooked at 81 °C for 10 h).

The formulation of the ED is shown in Table 1 and Table 2, which also show the formulations of both CM and RM. The chemical compositions of ED, CM and RM are shown in Table 3 and Table 4. The crude fat was analyzed by a fatty analyzer (FT640, Guangzhou, Grand Analytical Instrument Co., Ltd., Guangzhou, China) according to GB/T 6433-2006 (B type) [8]. The amount of crude protein was determined by using the Kjeldahl method with a semiautomatic Kjeldahl apparatus (VAPODEST 200, C. Gerhardt GmbH and Co., KG, Bonn, Germany) and following GB/T 6432-2018/7.2 [9]. The crude fiber was analyzed according to GB/T 6434-2006 [10]. The crude ash was analyzed according to GB/T 6438-2007 [11]. The starch was analyzed according to GB5009.9-2016/second method [12]. The moisture was analyzed according to GB/T 6435-2014/8.1 [13]. Ca was analyzed according to GB/T 6436-2018/4 [14]. P was analyzed according to GB/T 6437-2018 [15]. Mg was analyzed according to GB/T 13885-2017 [16]. Zn was analyzed according to GB/T 13885-2017 [16]. The taurine was analyzed according to GB/T 5009.169-2016/first method [17]. The Lys was analyzed according to GB/T 18246-2019 [18]. Diet was calculated for gross energy using the following formula (NRC, 2006):

$$Gross\;energy\;(kcal/kg)=\left(crude\;protein\;(kg)\times 5.7+crude\;fat\;(kg)\times 9.4+(carbohydrate\;(kg)+crude\;fiber\;(kg))\times 4.1\right)$$

Throughout the whole period of 28 days, all Ragdoll cats were provided with sufficient water and food (with twice the feeding amount that met their energy requirements calculated approximately, in accordance with the National Research Council (NRC, 2006) standard, making sure water and food were left over the next day) at 8 a.m. everyday.

### 2.2. Animals and Management

Fifteen healthy male Ragdoll cats, aged five months with an average weight of 2.92 ± 0.40 kg, were included in the study (all the cats were vaccinated before the experiment). According to the requirements of the feeding experiment, the cats were assigned to three treatment groups (*n* = 5/group) by initial body weight: fed ED, CM and RM. Cats were offered food at 8 a.m. daily. Before the experiment began, all the cats were fed extruded dry food, and after a 5-day transition period, they were gradually transferred to the corresponding experimental diet. Each cat was housed in a cat cage measuring 80 cm × 70 cm × 60 cm and furnished with a feeding bowl, a drinking bowl, and a litter box. The experiment lasted 28 days. The animal room was illuminated with artificial light, and the testing facility was equipped with a humidifier and air conditioner, maintaining a controlled temperature of 26 °C and a humidity of 60%. Throughout the experiment, the behavior of the Ragdoll cats was monitored daily, and their litter boxes were cleaned when they were dirty. All experiments were approved by the Animal Ethics Committee of Zhejiang University, AP CODE:ZJU20220501.

### 2.3. Growth Performance

At the start and end of the experiment, the cats were weighed at 8:00 a.m. with food and water access discontinued at 8:00 p.m. the day prior to the assessment (because the cats did not show any stress response, and the conditions of the three groups of cats were consistently relatively stable), and the initial and final body weights of the cats were recorded to calculate the average daily gain (ADG).

The daily feeding amount and remaining feed amount of each Ragdoll cat were recorded at 8 a.m. everyday, and the average daily feed intake of each age group was calculated.

### 2.4. Fecal Score

Stool samples were scored everyday at 8 a.m. according to the following criteria:

One point: The feces is dry and hard, usually excreted as single pellets, and is not loose on the ground.

Two points: The feces is dry but not firm, with visible segments in appearance, and a small amount of residue is left when the feces is removed from the ground.

Three points: The feces is long and columnar, with a wet surface and no or few segmentations in appearance; residues are picked up from the ground, but the shape is maintained. This is the optimal fecal score.

Four points: The feces is very wet, long, and columnar, with residues when picked up from the ground, and is unable to maintain its shape.

Five points: The feces is very wet, the shape can be observed, the shape is generally piled rather than columnar, there are residues when picked up from the ground, and the shape cannot be maintained.

Six points: The fecal structure can be observed, no shape is formed, the shape is pile-like or point-like, and it leaves residues when it is picked up from the ground.

Seven points: The feces appears as a water sample with no visible fecal structure and no formed shape, in a pool of water stains.

### 2.5. Sampling Collection

We collected all the samples on the last day of the experiment (28th day). Harvested hair samples (back hair with comb) from each group of each Ragdoll cats weighing about 1 g were stored at −20 °C for future use. An amount of 2 mL of whole blood was extracted from the leg vein and transferred to disodium EDTA anticoagulation tubes, mixed thoroughly, refrigerated, and promptly sent for analysis after sampling. Blood sample collection was facilitated by the School of Veterinary Medicine Affiliated with Zhejiang University. Additionally, 3 mL of whole blood sampled from the leg vein was allowed to stand for 30 min and was subsequently centrifuged at 3500 rpm for 15 min to separate the serum. The supernatant was transferred to a 1.5 mL centrifuge tube, which was rapidly frozen in liquid nitrogen and stored at −80 °C for later use. Whole feces for each cat was collected, and 10% HCl was added to the nitrogen fixation on days 25–28 at 8 a.m. Feces samples were stored at 20 °C, oven-dried at 65 °C for 48 h and finely ground to pass through a 1 mm mesh screen for future use [19].

### 2.6. Determination of Apparent Digestibility of Nutrients

During the experiment, feces from the last four days was collected using the whole feces method. The feed intake was recorded, and the levels of crude protein and crude fat in the feces were measured.

### 2.7. Determination of Blood Biochemical Parameters

Whole blood counts were conducted using a 5-class fully automated cytometer (IDEXX ProCyte Dx, IDEXX laboratories, Westbrook, America). The parameters assessed included white blood cell (WBC) count, lymphocyte (LYM) count, monocyte count (Mon), eosinophil count (Eos), basophil count (BASO), neutrophil count (NEUT), hemoglobin concentration (HGB), red blood cell (RBC) count, mean corpuscular volume (MCV), mean hemoglobin (MCH), mean hemoglobin concentration (MCHC), and plateletcrit (PCT), among others. Biochemical indicators in serum samples, such as total protein (TP), globulin-albumin (GLOB-ALB), glucose (GLU), urea nitrogen (UN), aspartate aminotransferase (AST) activity, alanine aminotransferase (ALT) activity, total cholesterol (TCHO), alkaline phosphatase (ALP), and creatinine (CRE), were analyzed using an automatic serum biochemistry analyzer (Hitachi Limited 7020, Hitachi High Tech, Tokyo, Japan) with kits obtained from Biosino Biotechnology and Science, Inc. (Beijing, China).

### 2.8. Determination of Antioxidant Indices

Serum antioxidant indices, including total superoxide dismutase (T-SOD), malondialdehyde (MDA), and glutathione peroxidase (GSH-Px) levels, were measured. All parameters were assessed using kits (Nanjing Jiancheng Bioengineering Institute, Nanjing, China), following their instructions and methods.

### 2.9. Electron Microscopic Observation of Hair

Hair samples from the sample bag were randomly selected and fixed on the operating table. After gold coating, the samples were observed under a scanning electron microscope (Chinese Academy of Sciences Scientific Instrument Factory, Beijin, China, KYKY-AMRAY-1000B) at magnifications of 1000× and 3000×.

### 2.10. Fecal 16S rRNA High-Throughput Sequencing

The genomic DNA of the fecal samples was extracted using the cetyltrimethylammonium bromide method and used for 16S rRNA sequencing. It was processed and analyzed by Beijing Novogene Co., Ltd. (Beijing, China). Sequencing libraries were generated using TruSeq R DNA PCR-Free Sample Preparation Kit (Illumina, San Diego, CA, USA) following the manufacturer’s recommendations, and index codes were added. The library’s quality was assessed on Qubit@2.0 Fluorometer (Thermo Scientific, Waltham, MA, USA) and in the Agilent Bioanalyzer 2100 system (Agilent, Santa Clara, CA, USA). At last, the library was sequenced on an Illumina NovaSeq platform, and 250 bp paired-end reads were generated. Then, the reads were filtered using QIIME quality filters (QIIME1.9.1) [1].

### 2.11. Statistical Analysis

Data analysis was performed using one-way ANOVA in SPSS 22.0 software (IBM, Armonk, NY, USA) for significance testing, and Duncan’s method was used for multiple comparisons. The data are presented as the mean ± standard deviation (Mean ± SD). One-way analysis of variance (ANOVA), followed by a least significant difference (LSD) multiple-range test, was used to determine the statistical significance of multiple comparisons in the experiment. For gut microbiota, operational taxonomy units (OTUs) with a similarity ≥97% were chosen for α-diversity analysis, and principal coordinate analysis (PCoA) and a box plot were used to evaluate the β-diversity.

## 3. Results

### 3.1. Performance

As shown in Table 5, there was no significant difference in body weight among the ED group, CM group, and RM group before and after the test (*p* > 0.05). However, the daily feed energy intake of the ED group was significantly higher than that of the CM and RM groups (*p* < 0.05), while there was no significant difference between the CM group and the RM group (*p* > 0.05). The ED group exhibited significantly higher fecal scores than did the CM and RM groups (*p* < 0.05), with no significant differences observed between the CM group and the RM group (*p* > 0.05).

As shown in Table 6, on Day 28, both the CM group and the RM group showed a significant increase in dry matter digestibility compared to the ED group (*p* < 0.05). Compared to the 5.34% increase in dry matter digestibility in the RM group, the CM group had a greater increase of 11.64%. Similarly, protein and fat digestibility were significantly greater in both meat groups than in the ED group (*p* < 0.05), with significant differences in protein digestibility observed between the CM and RM groups.

### 3.2. Hair Quality

**Figure 1:**
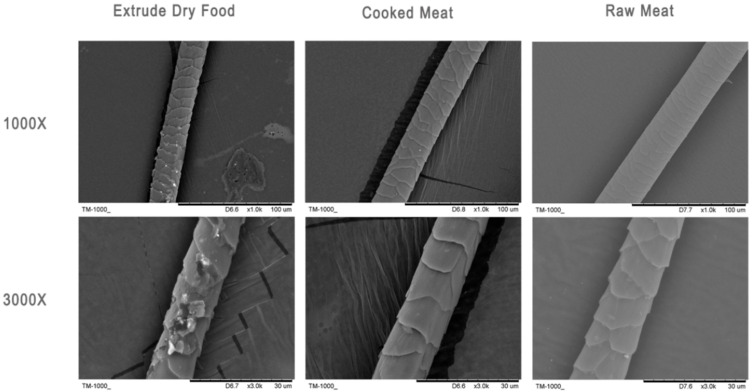
Scanning electron microscopy observations of Ragdoll cat hair.

As depicted in Figure 1, after 28 days of feeding, the cat hair scales in the ED group were damaged, showing a blurry and irregular surface structure, and the scales were not visible. Conversely, the CM group and RM group displayed intact scale structures, smooth surfaces, and clearly visible scale layers, which indicated superior hair quality compared to that of the ED group.

### 3.3. Whole Blood Count, Serum Biochemistry and Antioxidant Capacity

Table 7 demonstrates that there was no notable difference in the whole blood counts of cats on the three diets (*p* > 0.05).

As shown in Table 8, on Day 28, both the CM group and the RM group displayed a significant increase in globulin compared to the ED group (*p* < 0.05), with a significant difference observed between the CM and RM groups.

Table 9 shows that on Day 28, both the CM group and the RM group exhibited a significant increase in SOD and a significant decrease in MDA compared to the ED group (*p* < 0.05), with no significant differences between the meat groups (*p* > 0.05).

### 3.4. The Fecal Microbiota

**Figure 2:**
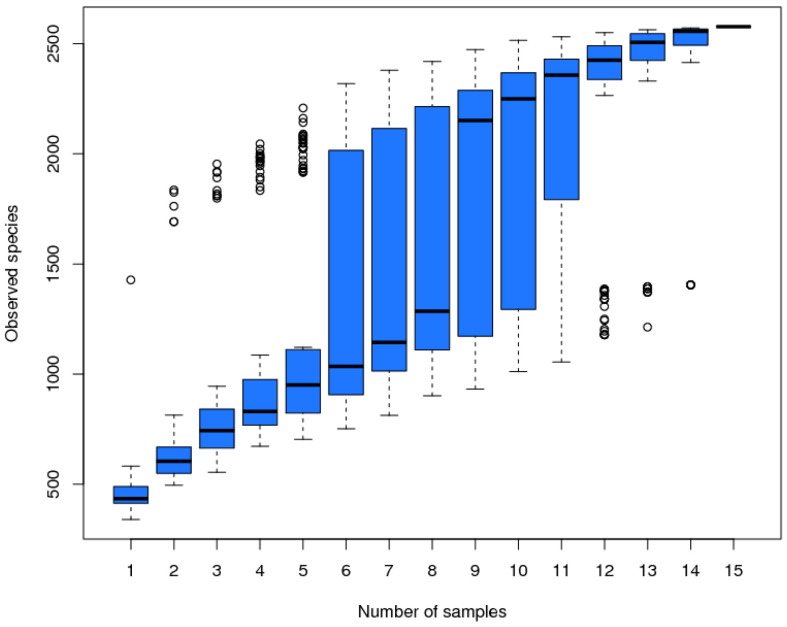
The species accumulation box plot of the three groups of samples 1–5 represents ED group, that of samples 6–10 represents the CM group, and that of samples 11–15 represents the RM group.

From Figure 2, it can be seen that the box plot tends to flatten out; therefore, the experimental samples were representative, which can be further analyzed.

**Figure 3:**
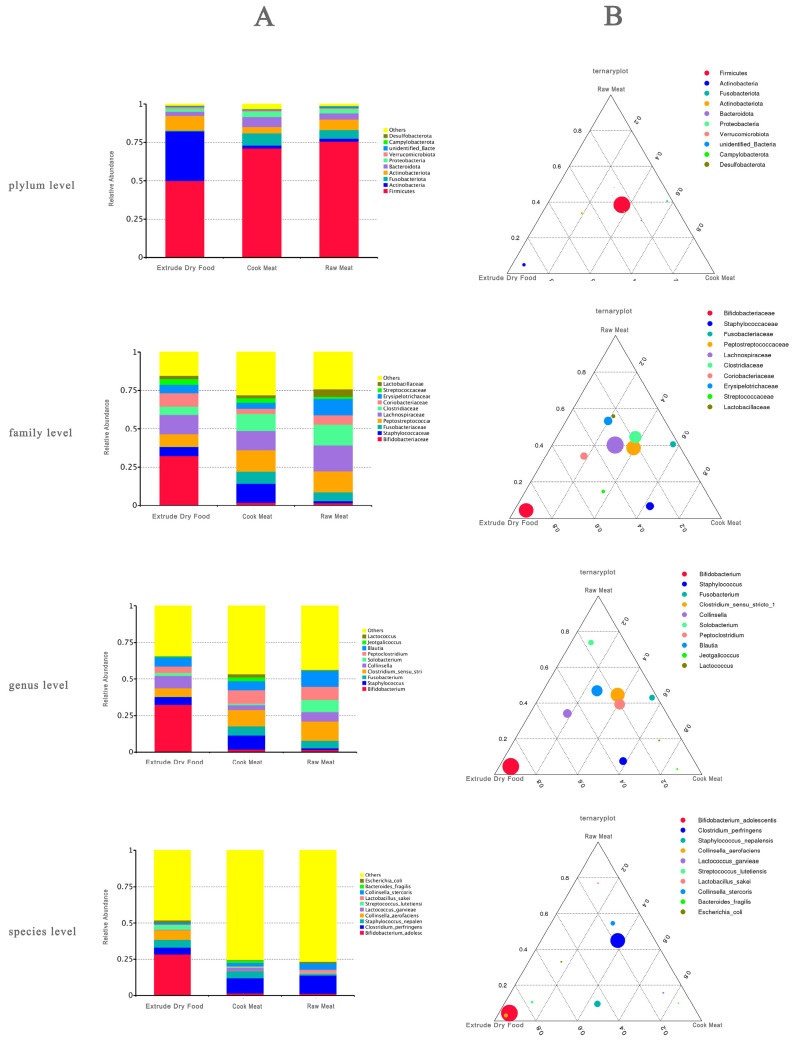
(**A**) Relative abundance of different-level species; (**B**) dominant species in each group of different-level species.

According to Figure 3, the predominant species in the ED group included Firmicutes, *Bifidobacterium*, and *Bifidobacterium adolescentis*, while those in the CM group included Firmicutes, Digestive Streptococcus, *Clostridium*, and *Clostridium perfringens*, and those in the RM group included Firmicutes, Lachnospira, *Clostridium*, and *Clostridium perfringens* as the dominant species.

**Figure 4:**
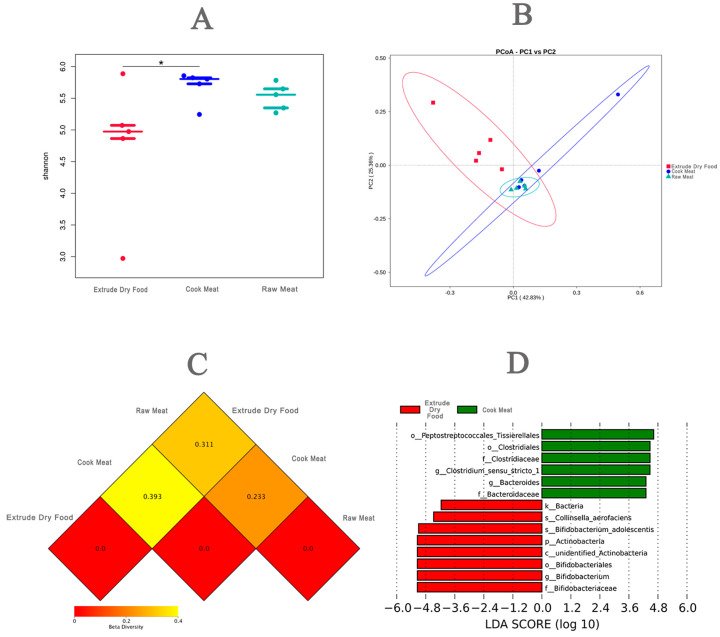
(**A**) Shannon–beeswarm curve; (**B**) PCoA analysis; (**C**) thermal diagram of coefficient of difference between groups; (**D**) microorganism difference between ED group and CM group, * *p* < 0.05.

Assessment of fecal alpha-diversity indices suggested that the Shannon index tended to be higher in the CM group and RM group than in the CON group on Day 28 (Figure 4A, *p* < 0.05). From the differences in the beta-diversity index based on weighted UniFrac distances, PCoA plots revealed distinct separation between the CON and meat diet groups on Day 28 (Figure 4B, *p* < 0.05).

Both cooked and RM diets exhibited similar effects on the fecal flora, markedly differing from those of ED (Figure 4C). Notably, both diets decreased the relative abundance of Bifidobacterium adolescentis, with Clostridium perfringens emerging as the predominant species in the fecal flora (Figure 4D).

## 4. Discussion

The three diets had no significant effect on the body weight of the Ragdoll cats, suggesting that they contributed similarly to maintaining the body weight of the Ragdoll cats, which is consistent with the results of previous studies on feline animals fed different diets [17]. The ED group had significantly higher average energy intake than the meat diet groups. Most animals regulated feed intake via the energy in food, which may seem contradictory to the results. However, it can be seen from Table 7 that the meat diet groups had significantly higher apparent digestibility of nutrients than the ED group, which may explain the occurrence of this phenomenon. Regarding stool consistency, the feces of the ED group was relatively soft, while that of the CM and RM groups fell within the normal range. We believe that a soft fecal state reveals a relatively unhealthy state of the animal intestine, which may be caused by disturbances to the intestinal bacteria [2].

CM exhibited the highest dry matter digestibility, crude protein digestibility, and fat digestibility, indicating its superior digestibility. Conversely, the ED group displayed the lowest dry matter digestibility, crude protein digestibility, and crude fat digestibility, consistent with the findings of Kerr et al. In their study, felines fed a ground adult chicken product (kind of CM) had higher nutrition digestibility than those fed extruded dry food. A previous study suggested that cooking provides benefits over raw chicken because of the greater AA availability it allows for [20]. We believe that this is because proteins and fats undergo hydrolysis into smaller molecular substances, facilitating digestion and absorption. This outcome suggests that pet owners who opt for wet food can appropriately reduce feeding amounts to mitigate excessive weight gain and prevent associated health risks. Furthermore, the digestibility of RM was lower than that of CM, which may contradict the idea that cats will have higher digestibility on a raw meat diet.

CM has the most pronounced immune-enhancing effect on the body. In a study, cats fed a raw diet showed a significant increase in lymphocyte and immunoglobulin production, whereas there were no significant changes for cats fed a cooked commercial moist diet [21]. This is somewhat different from our experimental results, and we speculate that the reason is due to differences in the processing temperature and cooking time for the CM diet.

Feeding Ragdoll cats CM or RM had a superior effect on their antioxidative capacity compared to feeding them ED. However, both groups demonstrated better antioxidative ability than that of the ED group. The results obtained from this study are consistent with the findings of a previous study showing that a meat diet indeed enhances antioxidant capacity [22].

Notably, the hair quality of the RM and CM groups showed considerable improvement compared to that of the ED group. Specifically, the hair cuticles of the CM and RM groups appeared smoother and exhibited a shinier appearance than those of the ED group. It has been reported in minks that the nourishing effects of diets on hair are due to their high Zn content [23,24]. We can see from Table 6 that meat diets have far more Zn than ED diets do, which is probably the reason for the difference between the three groups. We also speculate that it is possible that the higher digestibility of meat diets leads to a higher bioavailability of fatty acids, which benefits fur quality [25].

In this study, 16S rRNA sequencing analysis of fecal microbes from Ragdoll cats fed three diets revealed distinct effects on the fecal microbiome. Notably, the composition of fecal microbes in the ED group significantly differed from that in the other two groups, whereas the CM and RM groups shared similar gut microbial compositions. This study revealed that Firmicutes were the dominant species in all three groups of cats, consistent with previous studies on the gut microbiota in feline animals with different proportions of this species [26]. Further analysis revealed that both the CM and RM diets led to alterations in the microbial structure of the fecal flora at the genus level compared to that of the ED group. Specifically, there was a decrease in the relative abundance of *Bifidobacterium* and an increase in the relative abundance of *Clostridium*. Previous research has demonstrated that factors such as diet can influence the relative abundance of intestinal microorganisms such as Bifidobacterium and Clostridium, which may directly impact cholesterol metabolism and hyperlipidemia [27]. *Bifidobacterium adolescentis*, a representative member of the normal intestinal flora in mammals, is recognized as an intestinal probiotic that is crucial for maintaining intestinal homeostasis and microbial balance [26]. Our 16S rRNA sequencing analysis revealed that ED increased the relative abundance of *Bifidobacterium adolescentis*, suggesting its beneficial role in maintaining a healthy intestinal microbiota.

Given that existing flora analyses primarily focus on humans or economically important animals and overlook carnivores, a comprehensive assessment of the pros and cons of the three diets concerning the impact of the dominant species on the fecal flora of Ragdoll cats is warranted. Although *Clostridium perfringens* is a common component of the normal gastrointestinal flora, it is also recognized as a potentially harmful intestinal bacterium due to its ability to cause anaerobic infections and produce toxins [28]. The promotion of *Clostridium perfringens* dominance by both CM and RM diets may disrupt the normal structure of the fecal flora. A previous study suggested that the proportion of *Clostridium perfringens* in the gut of diarrheal cats is increasing [29]. Interestingly, in this study, we found that cats fed CM and RM had lower fecal scores than those in the ED group (*p* < 0.05), which is inconsistent with previous research findings. Another study reported that the amount of *Clostridium perfringens* had no impact on cats with GI disease [30]. However, recent studies have shed light on the involvement of the Clostridium family, particularly *Clostridium perfringens*, in the butyrate synthesis pathway from proteins in carnivores [31]. Butyrate, a type of SCFA, can directly act on immune cells in the intestinal mucosa, increase the number and activity of regulatory T cells (Tregs), and inhibit the activity of neutrophils, macrophages, dendritic cells and effector T cells [32]. One study showed that the Clostridium family could significantly increase the contents of acetic acid and butyric acid in the feces of pet cats, which may be one of the important factors for reducing the incidence of soft stool and diarrhea in pet cats [33]. Another Clostridium bacterium, *F. varium*, known for its ability to produce butyrate from protein sources, was found to be more abundant in dogs fed an RM-based diet for an extended period [34]. This suggests that the microbiome adapts to a long-term raw meat diet [25]. Moreover, members of the Clostridium family have been observed to be more prevalent in other carnivore species, such as cats and wolves [30,35,36]. Considering that the main difference between puffed dry food and meat is starch content, we speculate that starch-based raw materials are more beneficial for the proliferation of bifidobacteria, while a pure meat diet increases the abundance of bacteria in the normal gut microbiota of feline animals, namely Clostridium perfringens. Similar to our study, all of the previously mentioned researchers reported a greater relative abundance of fecal Fusobacteria in dogs fed RM than in those fed ED [37,38].

However, in order to properly assess the impact of pet food processing, diets should have similar nutritional compositions. When expressed as dry matter, there was a significant difference in macronutrient content between groups on the ED diet and the meat diet due to process limitations, which was a study limitation of this experiment.

## 5. Conclusions

Our study revealed that different processed diets affect the metabolic health of Ragdoll cats. CM allowed for greater nutritional digestibility in purebred Ragdoll cats, enhances their immune and antioxidant capabilities, and makes their hair smoother. Meat diets increased the innate gut microbiota of felines, such as Clostridium, which may be more beneficial for their gut health as carnivores. These data, to some extent, suggest that CM is the most suitable diet for Ragdoll cats. Our research findings are beneficial for providing better theoretical support for pet cat breeders to choose between the different types of pet diets.

## Figures and Tables

**Table 1:** Formulation of ED diets.

Composition	Proportion (%)
Chicken	25.00
Egg	5.00
Chicken liver	5.00
Flatfish	4.00
Herring	4.00
Chicken neck	4.00
Dehydrated chicken	8.00
Fish meal	12.00
Chicken oil	3.00
Fish oil	1.00
Vegetable	1.00

ED: extruded dry food.

**Table 2:** Formulation of meat diets.

Composition	Proportion (%)
Chicken	40.00
Duck	40.00
Chicken heart	4.00
Chicken liver	5.00
Duck bone	9.00
Lucerne	1.00
Premix ^1^	1.00

^1^ Provided per kilogram of diet: vitamin A, 25,000.00 IU; vitamin D, 2200.00 IU; vitamin E, 25.00 mg; vitamin B1, 8.00 mg; vitamin B2, 11.00 mg; vitamin B6, 6.50 mg; vitamin B12, 0.06 mg; nicotinic acid, 55.00 mg; calcium pantothenate, 3.3 mg; D-biotin, 0.65 mg; folic acid, 1.00 mg; cholinechloride, 2600.80 mg; Fe, 100.00 mg; Cu, 10.00 mg; Mn, 1.50 mg; Zn, 15.00 mg; I, 1.50 mg; and Se, 0.20 mg.

**Table 3:** Chemical composition of ED diets in wet matter.

Nutritional Composition	ED	CM	RM
GE (kcal/kg)	5006.83	1759.20	1763.15
Crude protein (%)	45.60	22.00	22.03
Crude fat (%)	17.40	5.20	5.25
Crude fiber (%)	0.83	0.20	0.19
Crude Ash (%)	9.60	2.50	2.47
Starch (%)	18.00	0.20	0.15
Moisture (%)	8.47	68.4	68.3
Ca (%)	2.30	1.30	1.31
P (%)	1.80	1.10	1.13
Mg (%)	0.13	0.040	0.038
Zn (mg/kg)	220.00	227	218
Taurine (%)	0.18	1.00	1.05
Lys (%)	2.30	1.80	1.83

The GE (gross energy) was calculated, and the other values were analyzed. ED: extruded dry food. CM: cooked meat. RM: raw meat.

**Table 4:** Chemical composition of dry matter in different diets.

Composition	ED	CM	RM
Crude protein (%)	49.82	69.62	69.72
Crude fat (%)	19.01	16.45	16.61
Crude fiber (%)	0.91	0.63	0.61
Crude Ash (%)	10.49	7.91	7.82
Starch (%)	19.67	0.63	0.47
Ca (%)	2.51	4.11	4.15
P (%)	1.97	3.48	3.57
Mg (%)	0.14	0.13	0.12
Zn (mg/kg)	240.36	718.32	689.87
Taurine (%)	0.20	3.16	3.32
Lys (%)	2.51	5.70	5.79

ED: extruded dry food. CM: cooked meat. RM: raw meat.

**Table 5:** Effect of different diet patterns on the growth indices of Ragdoll cats.

Item	ED	CM	RM	*p*-Value
Initial weight (kg)	2.94 ± 0.43	2.93 ± 0.46	2.90 ± 0.40	0.990
Final weight (kg)	3.54 ± 0.30	3.55 ± 0.36	3.58 ± 0.33	0.974
Average daily gain (kg/d)	0.02 ± 0.00	0.02 ± 0.00	0.02 ± 0.00	0.628
Average feed intake (g/d)	68.60 ± 7.54 ^b^	169.20 ± 10.99 ^a^	168.00 ± 6.67 ^a^	<0.001
Average energy intake (g/d)	343.47 ± 37.37 ^a^	297.65 ± 19.33 ^b^	295.54 ± 11.74 ^b^	<0.001
fecal score	4.58 ± 0.37 ^a^	3.58 ± 0.11 ^b^	3.54 ± 0.36 ^b^	<0.001

^a,b^ Means within the same row with different superscripts differ significantly (*p* < 0.05). The same follows. ED: extruded dry food. CM: cooked meat. RM: raw meat.

**Table 6:** Digestibility of different processed diets for Ragdoll cats (tested at the end of the experiment).

Item	ED	CM	RM	*p*-Value
Dry matter digestibility (%)	81.45 ± 2.13 ^c^	90.93 ± 1.76 ^a^	85.80 ± 2.68 ^b^	<0.001
Crude protein digestibility (%)	89.97 ± 1.46 ^c^	96.58 ± 0.66 ^a^	94.67 ± 1.10 ^b^	<0.001
Crude fat digestibility (%)	92.93 ± 1.04 ^b^	96.33 ± 0.78 ^a^	95.28 ± 0.58 ^a^	<0.001

ED means extruded dry food, CM means cooked meat, RM means raw meat. ^a,b,c^ Values in a row with no common superscripts differ significantly (*p* < 0.05).

**Table 7:** Effects of different diets on the blood parameters of Ragdoll cats.

Item	ED	CM	RM	*p*-Value
Red blood cell count (M/μL)	9.26 ± 1.48	10.25 ± 0.93	10.36 ± 0.93	0.287
Hemoglobin concentration (g/dL)	13.42 ± 2.16	13.92 ± 1.23	14.40 ± 1.53	0.665
Mean erythrocyte hemoglobin concentration (g/dL)	35.44 ± 1.31	35.00 ± 0.85	34.68 ± 0.85	0.520
Red blood cell distribution width (%)	25.98 ± 1.53	27.92 ± 1.42	27.32 ± 1.16	0.115
Hematocrit (%)	37.88 ± 6.14	39.84 ± 4.01	41.52 ± 3.90	0.506
Mean red blood cell volume (fL)	40.90 ± 1.31	38.86 ± 1.53	40.08 ± 1.00	0.081
White blood cell count (K/μL)	15.52 ± 5.99	14.12 ± 6.04	14.55 ± 3.96	0.916
Lymphocyte count (K/μL)	4.01 ± 2.54	3.73 ± 0.88	4.61 ± 1.08	0.705
Monocyte count (K/μL)	0.53 ± 0.26	0.57 ± 0.23	0.64 ± 0.22	0.747
Total basophils (K/μL)	0.11 ± 0.07	0.10 ± 0.03	0.10 ± 0.05	0.869
Total eosinophils (K/μL)	0.84 ± 0.08	1.06 ± 0.40	1.00 ± 0.14	0.616
Total number of neutrophils (K/μL)	7.49 ± 2.24	7.52 ± 1.48	7.53 ± 2.92	0.826
Mean platelet volume (fL)	14.08 ± 2.00	14.60 ± 1.18	15.84 ± 2.30	0.350

ED means extruded dry food, CM means cooked meat, RM means raw meat. Mean values are based on one kitten per replicate and 5 replicates per treatment.

**Table 8:** Effect of the different diets on the serum biochemical indicators of Ragdoll cats.

Item	ED	CM	RM	*p*-Value
Glucose/(mmol/L)	10.07 ± 1.03	10.47 ± 0.66	9.75 ± 0.43	0.345
Triglycerides/(mmol/L)	0.11 ± 0.03	0.11 ± 0.04	0.11 ± 0.03	0.995
Total cholesterol/(mmol/L)	2.05 ± 0.17	1.858 ± 0.08	1.974 ± 0.21	0.203
Serum total protein/(g/L)	57.24 ± 7.55	62.55 ± 7.38	62.44 ± 11.48	0.581
Aspartate aminotransferase/(U/L)	17.36 ± 1.72	16.04 ± 1.16	17.40 ± 1.47	0.287
Alanine aminotransferase/(U/L)	19.53 ± 0.90	19.40 ± 1.40	20.11 ± 1.78	0.706
Urea nitrogen/(mmol/L)	0.80 ± 0.14	0.82 ± 0.88	0.80 ± 0.09	0.934
Alkaline phosphatase/(U/L)	30.41 ± 2.29	32.56 ± 1.61	32.28 ± 3.76	0.998
Globulin/(g/L)	30.33 ± 1.84 ^c^	36.20 ± 1.20 ^a^	33.89 ± 1.80 ^b^	<0.001
Albumin/(g/L)	26.91 ± 7.44	24.36 ± 7.32	28.54 ± 11.24	0.921
Creatinine/(mg/dL)	1.36 ± 0.07	1.36 ± 0.14	1.38 ± 0.11	0.958

ED means extruded dry food, CM means cooked meat, RM means raw meat. ^a,b,c^ Values in a row with no common superscripts differ significantly (*p* < 0.05).

**Table 9:** Effects of different diets on the serum antioxidants of Ragdoll cats.

Item	ED	CM	RM	*p*-Value
SOD/(U/mL)	25.54 ± 1.22 ^b^	29.72 ± 1.04 ^a^	28.62 ± 0.87 ^a^	<0.001
GSH/-PX (U/mL)	1591.30 ± 103.20	1724.97 ± 81.01	1719.51 ± 119.43	0.106
MDA/(nmol/mL)	6.35 ± 0.71 ^a^	5.44 ± 0.18 ^b^	5.29 ± 0.06 ^b^	0.004

ED, extruded dry food; CM, cooked meat; RM, raw meat. SOD, superoxide dismutase; MDA, malondialdehyde; GSH-PX, glutathione peroxidase. ^a,b^ Values in a row with no common superscripts differ significantly (*p* < 0.05).

## Data Availability

The original contributions presented in the study are included in the article; further inquiries can be directed to the corresponding author.
